# The effectiveness of moxibustion for the treatment of functional constipation: a randomized, sham-controlled, patient blinded, pilot clinical trial

**DOI:** 10.1186/1472-6882-11-124

**Published:** 2011-12-02

**Authors:** Ji-Eun Park, Jae-Uk Sul, Kyungwon Kang, Byung-Cheul Shin, Kwon-Eui Hong, Sun-Mi Choi

**Affiliations:** 1 Department of Medical Research, Korea Institute of Oriental Medicine, Daejeon, South Korea; 2 Korean Medicine Hospital, Pusan National University, Yangsan, South Korea; 3 Division of Clinical Medicine, School of Korean Medicine, Pusan National University, Yangsan, South Korea; 4 Department of Oriental Medicine, Daejeon University, Daejeon, South Korea

## Abstract

**Background:**

Moxibustion is an ancient traditional medicine using burning mugworts to stimulate acupuncture points. The aim of this study was to investigate the safety and efficacy of moxibustion for the treatment of constipation using a randomized, sham-controlled, participant-blinded, pilot trial.

**Methods:**

Twenty-six participants (identified with either qi (vital energy) deficiency or qi excess syndrome) were randomly divided into either a moxibustion or sham group. Participants were treated with real or sham moxibustion at 4 acupuncture points, ST23 and ST27, bilaterally, 3 times per week for four weeks. The primary outcome was the frequency of defecations; secondary outcomes were the Bristol stool form scale (BSS) and the constipation assessment scale (CAS).

**Results:**

Of the 26 participants that were randomized, 24 completed the study. Defecation frequency, BSS, and CAS showed no difference between the moxibustion and sham groups. The differences were -0.25 (95% CI: -2.08, 1.58, p = 0.78), -1.22 (95% CI: -2.7, 0.26, p = 0.1), 0.91 (95% CI: -1.46, 3.28, p = 0.44) in defecation frequency, BSS, CAS, respectively. The defecation frequency increased from an average of 3.3 to 4.6 times per week in the moxibustion group (1.5[-0.5, 2], *p *= 0.06) and from 2.7 to 3.7 stools per week in the sham group (1[-1, 2], *p *= 0.15) after four weeks of treatment. The difference between participants with a deficiency or an excess syndrome, determined based on assessment of sweat, facial features, pain, body energy, and pulse type, was significant in only defecation frequency. The difference was 3.3 (95% CI: 0.41, 6.19, *p *= 0.03).

**Conclusion:**

Moxibustion treatment appears safe, but showed no positive effect on constipation. The effectiveness of moxibustion treatment may depend on the syndrome pattern, and further long-term studies with a larger number of subjects are warranted.

**Trial registration:**

Clinical Research Information Service, KCT0000168

## Background

Constipation is a common gastrointestinal complaint that is experienced by 27.2% of Canadians [1], 12%-19% of Americans [2], and 14% of Asians [3], with a prevalence of 11.6% in the elderly Asian [4]. In Korea, the prevalence is 16.5% for self-reported constipation, 9.2% for functional constipation and 3.9% for constipation-predominant irritable bowel syndrome [5].

Constipation can cause abdominal pain, discomfort, gas, headache, nausea, and anorexia as well as potentially contributing to functional loss and length of stay in the hospital [6]. Patients spend approximately $7,900 a year on health care costs for their constipation [7]. However, many patients are disappointed by current conventional treatments and seek help from complementary and alternative medicine practitioners [8].

Moxibustion has been widely used in Asian countries as traditional Chinese medicine (TCM). Acupuncture and moxibustion are similar except that their stimulation methods are different. Moxibustion uses the heat generated by burning herbal preparations containing *Artemisia vulgaris *to stimulate acupuncture points. Moxibustion treatment, though uncommon in Western countries, has been shown to have benefits for pain [9], cancer care [10], and ulcerative colitis [11].

To date, there have been three randomized controlled clinical trials (RCTs) testing the effectiveness of moxibustion for the relief of constipation [12]. Previous RCTs have shown that moxibustion treatment was more effective against constipation than a glycerin enema [13,14] or no treatment [15]. However, the methodology used in these studies may have had a risk of bias [12], and a clinical trial comparing moxibustion with sham treatment does not exist.

The aims of this rigorous, pilot patient-blinded RCT are to evaluate the safety and efficacy of moxibustion for the treatment of constipation by comparing moxibustion and sham treatment.

## Methods

### Study design and ethics approval

This study was a single-center, randomized, parallel, sham-controlled, patient-blinded pilot clinical trial to evaluate the safety and efficacy of moxibustion in subjects with constipation in Korea. The Oriental Medical Doctor (OMD) initially screened each potential participant against the inclusion and exclusion criteria. After completing a screening test, participants entered a one-week baseline period without moxibustion treatment. At the end of the baseline period, eligible subjects were randomized to either the moxibustion or the sham group. The treatment period consisted of one week of baseline assessment, four weeks of treatment, and two weeks of follow-up, for a total study period of seven weeks.

All participants were blinded to the type of treatment received until completion of the study. To avoid allocation bias, concealed allocation using a sealed envelope was employed in this study. The study was conducted in accordance with the Declaration of Helsinki, and written informed consent was obtained from each participant before allocation. The study was approved by the Institutional Review Board of Daejeon University Hospital, Daejeon, South Korea, where the study took place. Full details of the trial protocol can be found at cris.cdc.go.kr [16].

### Inclusion/exclusion criteria

The recruitment of subjects took place from May to September in 2009. The study subjects consisted of adults aged 19 to 55 years with at least a six-month history of constipation according to the Rome criteria [17]. To be included in the study, participants had to have at least two of the following symptoms on more than 25% of occasions: straining; lumpy or hard stools; a sensation of incomplete evacuation; a sensation of anorectal obstruction/blockage; manual maneuvers to facilitate defecation; or defecation frequency less than three times per week.

Subjects were excluded from the study if they had inflammatory bowel disease or other structural bowel diseases, a diagnosis of irritable bowel syndrome (IBS), other significant disorders or diseases that may interfere with completion of the study, or used laxatives two weeks prior to the baseline assessment. Pregnant or breastfeeding women were excluded as were participants reporting more than one loose or watery stool each day during the baseline period. Magmil (Magnesium Hydroxide) was allowed as a rescue medication for participants who had intolerable discomfort due to severe constipation, but use was monitored.

### Recruitment and randomization procedures

Participants were recruited through advertisements in local newspapers and the hospital website and bulletin boards. Candidates were interviewed and evaluated to determine eligibility. Written informed consent was obtained from eligible candidates. A statistician randomized participants using computer-generated random table in a 1:1 ratio with block size 4, and clinical research coordinators (CRC) assigned them by the random table to receive real moxibustion or sham treatment.

### Treatments

Moxibustion or sham treatment was applied three times per week for four weeks (a total of 12 treatments). All moxibustion treatments were applied to acupuncture points at bilateral ST23 (Taiyi) and ST27 (Daju), as those sites were included in the Stomach Meridian of Foot Yangmyeong and have been shown to improve gastro-intestinal function such as dyspepsia, abdominal pain and constipation [18,19]. Four OMD, each having a national license and practical experience of more than 5 years, selected the acupuncture points, frequency of moxibustion, number of session through consensus among them after reviewing the major textbook of acupuncture and moxibustion [18-20]. Participants in the treatment group received moxibustion with a moxa pillar (0.6 × 20 mm, Kihoang company, South Korea) 3 times at each point in a single session.

**Figure 1:**
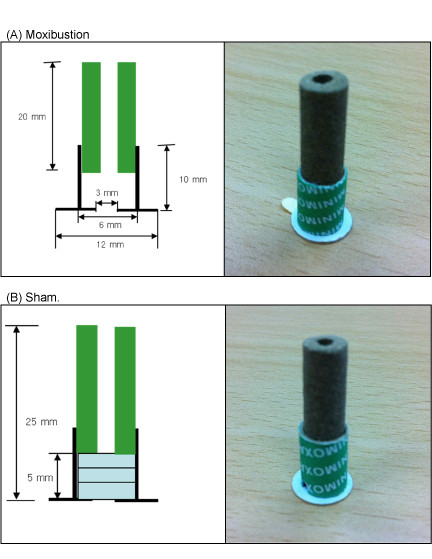
**Diagram and appearance of the moxibustion (A) and sham (B)**.

Sham treatment was given by adding insulation below the moxa pillar to prevent the transfer of heat from the moxa pillar to the patient. The sham treatment looks similar to the real moxibustion treatment in its appearance and burning procedure; therefore, participants were able to smell the smoke or observe the burning moxa (Figure 1). The validity of this method was well established as blinded to the participants in a previous study [21].

All moxibustion treatments were performed by the same experienced OMD practitioner who has had at least six years of training in acupuncture and moxibustion therapy.

### Assessment

The primary outcome was the change in the number of defecations per week from baseline to after the treatment period. Secondary outcomes included the Bristol Stool Form Scale (BSS) and the Constipation Assessment Scale (CAS). All assessments were conducted weekly by a researcher in each treatment arm.

BSS is designed to classify the form of stool into seven categories, from 'separate hard lumps, like nuts (Type 1)' to 'watery, no solid pieces (Type 7)' [22]. All participants had to record their defecation frequency and stool form in a diary format. At each visit, the diary was checked by an assessor and supplemented if further details were required.

The CAS is an eight-item tool that assesses the universal characteristics of constipation and assigns a score for constipation on a three point summated rating scale ranging from 0 'no problem' to 2 'severe problem'. The CAS has 8 items, therefore total CAS score has ranged from 0 to 16 [23]. The characteristics of constipation listed on this tool include 'abdominal distension or bloating', 'change in amount of gas passed rectally', 'less frequent bowel movement', 'oozing liquid stool', 'rectal fullness or pressure', 'rectal pain with bowel movement', 'small volume of stool' and 'being unable to pass stool'.

### Syndrome pattern differentiation of deficiency and excess syndromes

As constipation is considered to be caused by either a deficiency or excess of *qi *in traditional medicine in China and Korea, we developed questionnaires to differentiate between the deficiency and excess syndromes based on a validated previous study [24]. The questionnaire used in this study to determine whether a patient had a deficiency or an excess syndrome included an assessment of sweat, facial features, stomach pain, oppressive pain, stuffiness, body energy, duration of disease, and pulse type. A patient with a deficiency syndrome has sunken, weak pulse, whereas a patient with an excess syndrome has superficial and broad pulse. The patients having symptoms such as a pale face, heavy sweat, and depression were considered to have a deficiency syndrome; the patients having symptoms such as a swollen face, little sweat, and chest pressure were considered to have an excess syndrome. Syndrome pattern differentiation was conducted by an OMD before randomization.

### Statistical analysis

All data were entered into a data sheet twice and reviewed to ensure accuracy. The analyst (a statistician, KWK) was blinded to group allocation. Intent-to-treat (ITT) analyses were conducted; missing data were replaced with the last observation value carried forward (LOCF) method. One subject in the moxibustion group was dropped after randomization without any clinical data. Therefore, the ITT was 12 in the moxibustion group and 13 in the sham group. All data are summarized as mean ± standard deviation (SD) in continuous data and as frequency (%) in dichotomous data. An independent Student's t-test was used to analyze the baseline differences between groups and to evaluate the effect of treatment on the mean change in scores between baseline and week four for all continuous variables. Paired Student's t-test was used to analyze the differences between the values at baseline and week four for all continuous variables.

For sample size calculation, there was no previous clinical trial to compare the moxibustion with sham moxibustion, therefore, this study was designed as a pilot study to calculate the appropriate sample size for future rigorous randomized clinical trials. Each group was considered 13 participants as minimum sample size for evaluating the effect of moxibustion [25,26].

Statistical analyses were performed using the SAS statistical package (v.9.1, SAS institute, Inc., Cary, NC, USA), and the level of significance was established at *p *= 0.05.

## Results

### Demographic data

**Figure 2:**
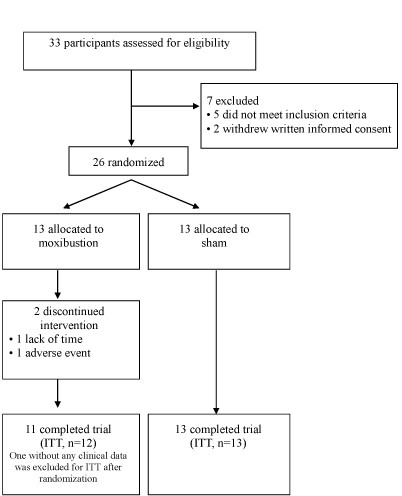
**CONSORT flow chart**.

Thirty-three were screened for inclusion in this study by CRCs from July to September 2009. Seven of these subjects were excluded; five did not meet the inclusion criteria (three had taken a prohibited drug, and 2 did not meet the functional constipation criteria) and two withdrew written informed consent. The remaining 26 participants were randomized into either the moxibustion group (n = 13) or the sham group (n = 13); 24 completed all treatments and the follow-up period. Two participants of the moxibustion group, withdrew without completing the study, one due to an adverse event and one due to an inability to complete all treatment (Figure 2). All of the participants were women. The demographic data were not significantly different between the moxibustion and sham groups (Table 1).

**Table 1:** Demographic data of moxibustion and sham groups at baseline

		Moxibustion (n = 12)	Sham (n = 13)	*p*-value
Sex (female (%))		12 (100)	13 (100)	1.00

Age (years)		36.15 ± 15.24	37.08 ± 11.58	0.65

Height (cm)		158.83 ± 3.76	158.0 ± 4.73	0.63

Weight (kg)		52.67 ± 3.26	59.15 ± 10.31	0.05

Temperature (°C)		36.58 ± 0.19	36.40 ± 0.70	0.40

Blood pressure (mm Hg)	Systolic	103.69 ± 10.56	109.77 ± 9.27	0.25
	
	Diastolic	64.27 ± 10.30	64.85 ± 7.72	0.88

Pulse (beats/min)		77.36 ± 9.18	72.92 ± 6.95	0.19

Stool frequency (number/week)		3.33 ± 1.61	2.69 ± 1.38	0.39

BSS		3.07 ± 1.13	3.97 ± 1.08	0.06

CAS		7.25 ± 3.55	8.08 ± 2.36	0.50

Values are mean ± standard deviation. BSS: Bristol stool form scale; CAS: constipation assessment scale.

*p*-value, by independent Student's t-test.

### Primary outcome variable

There was no significant difference in the change in defecation frequency between the moxibustion and sham groups. The difference taking the change of moxibustion group from that of sham group was -0.25 (95% CI: -2.08, 1.58, *p *= 0.78). Defecation frequency increased from an average of 3.3 to 4.6 times per week in the moxibustion group and from 2.7 to 3.7 stools per week in the sham group after four weeks of treatment.

**Figure 3:**
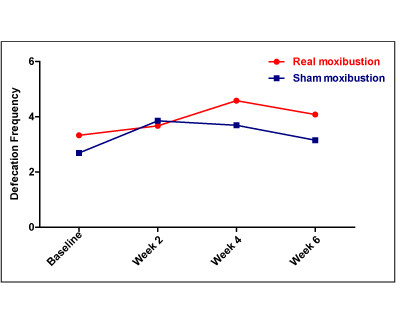
**Defecation frequency in moxibustion and sham groups**. All *p*-value > 0.05, by independent t-test.

The moxibustion group showed differences in defecation frequency that approached significance when compared to the baseline values (1.5[-0.5, 2], *p *= 0.06), but the sham group showed no significant difference after treatment (1[-1, 2], *p *= 0.15). (Figure 3, Table 2).

**Table 2:** Effects of treatment between moxibustion and sham groups

	Pre- and post-treatment											
	**Baseline**			**Week 2**			**Week 4**			**Week 6**		

	**Moxibustion** **(n = 12)**	**Sham** **(n = 13)**	***p***	**Moxibustion** **(n = 12)**	**Sham** **(n = 13)**	***p***	**Moxibustion** **(n = 12)**	**Sham** **(n = 13)**	***p***	**Moxibustion** **(n = 12)**	**Sham** **(n = 13)**	***p***

Defecation Frequency (times/week)	3.33 ± 1.61	2.69 ± 1.38	^†^0.39	3.67 ± 2.10	3.85 ± 1.68	0.38	4.58 ± 2.39	3.69 ± 1.93	0.78	4.08 ± 1.56	3.15 ± 1.34	^†^0.62

BSS	3.07 ± 1.13	3.97 ± 1.08	0.05	3.37 ± 1.19	4.36 ± 1.57	0.87	3.69 ± 1.29	3.36 ± 1.70	0.10	3.53 ± 1.25	3.86 ± 1.35	0.34

CAS	7.25 ± 3.55	8.08 ± 2.36	0.50	4.67 ± 3.20	3.85 ± 1.46	0.09	3.42 ± 2.15	5.15 ± 3.87	0.44	3.50 ± 3.90	4.85 ± 3.46	0.68

Values are mean ± standard deviation. BSS: Bristol stool form scale; CAS: constipation assessment scale.

*p*-value, by independent t-test.

^† ^analyzed by Wilcoxon rank-sum test

### Secondary outcome variables

The difference in the change in the BSS score between the moxibustion and sham group was -1.22 (95% CI: -2.7, 0.26, *p *= 0.1) in weeks 4, and -0.56 in weeks 6 (95% CI: -1.76, 0.64, *p *= 0.34). The difference in the CAS score between the two groups was 0.91 (95% CI: -1.46, 3.28, *p *= 0.44) in weeks 4, and 0.52 (95% CI: -2.02, 3.06, *p *= 0.68) in weeks 6 (Table 2).

However, when compared to baseline the moxibustion group had a significant change in BSS score after the 2 weeks of follow-up (0.45 [-0.04, 1], *p *= 0.048). The change in CAS from baseline was significant in both the moxibustion (-3 [-4.5, 0], p < .01 in 2 weeks, -4 [-5.5, -3], p < .001 in 4 weeks, and -4.5 [-6.5, -1], p < .01 in 6 weeks) and sham (-4 [-5, -3], p < 0.001 in 2 weeks, -4 [-4, -1], p < .01 in 4 weeks, -3 [-4, -1], p < .001 in follow-up) treatment groups.

### Syndrome pattern differentiation of deficiency and excess syndromes

Five participants were diagnosed with an excess syndrome, and twenty-one participants were diagnosed with a deficiency syndrome. In this study, the most prevalent symptoms for an excess syndrome were a strong body energy and superficial pulse; for a deficiency syndrome the symptoms were a long duration of disease and weak body energy.

Of the participants who received moxibustion treatment, ten of 13 were diagnosed with a deficiency syndrome. There were no significant differences between the participants with a deficiency or an excess syndrome at baseline in terms of the demographic data and constipation symptoms, including defecation frequency, BSS and CAS score.

**Figure 4:**
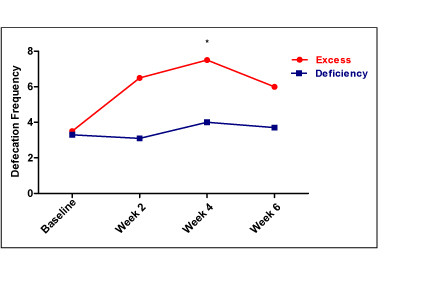
**Defecation frequency in moxibustion treatment between the excess and deficiency syndromes**. * *p = 0.03*, by independent t-test.

In the defecation frequency, moxibustion treatment showed significantly greater effect on participants with an excess syndrome than those with a deficiency syndrome. The difference was 3.3 (95% CI: 0.41, 6.19, p = 0.03). However, neither the BSS nor CAS scores were significantly different between these two groups after moxibustion treatment. The difference between two groups was 0.33 (95% CI: -1.54, 2.19, *p *= 0.70) in BSS and -1.4 (95% CI: -5.71, 2.91, *p *= 0.49) in CAS (Table 3, Figure 4).

**Table 3:** Effects of moxibustion treatment on patients with excess or deficiency syndrome patterns

	Pre- and post-treatment											
	**Baseline**			**Week 2**			**Week 4**			**Week 6**		

	**Excess** **(n = 2)**	**Deficiency** **(n = 10)**	***p***	**Excess** **(n = 2)**	**Deficiency** **(n = 10)**	***p***	**Excess** **(n = 2)**	**Deficiency** **(n = 10)**	***p***	**Excess** **(n = 2)**	**Deficiency** **(n = 10)**	***p***

Defecation Frequency (times/week)	3.50 ± 2.12	3.30 ± 1.64	^†^1.00	6.50 ± 0.71	3.10 ± 1.79	0.08	7.50 ± 0.71	4.0 ± 2.16	0.03	6.00 ± 1.41	3.70 ± 1.34	0.09

BSS	3.60 ± 1.98	2.97 ± 1.02	0.50	4.57 ± 2.22	3.13 ± 0.89	0.31	4.49 ± 2.31	3.53 ± 1.13	0.70	4.03 ± 1.66	3.43 ± 1.25	0.96

CAS	7.0 ± 5.66	7.3 ± 3.43	0.92	4.00 ± 5.66	4.80 ± 2.97	^†^0.92	2.00 ± 2.83	3.70 ± 2.06	0.49	3.50 ± 2.12	3.50 ± 4.25	0.92

Values are mean ± standard deviation. BSS: Bristol stool form scale; CAS: constipation assessment scale.

*p*-value, by independent t-test.

^† ^analyzed by Wilcoxon rank-sum test

### Safety

There were no serious adverse events reported during the study period. Two participants in the moxibustion group and one participant in the sham group reported rubefaction with or without an itching sensation. Only one patient in the moxibustion group dropped out due to this adverse event. All adverse events reported in this study were mild and transient.

## Discussion

Our study is the first rigorous randomized, sham-controlled, patient blinded clinical trial to evaluate the safety and efficacy of moxibustion for the treatment of constipation in adults. These study results suggest that moxibustion treatment showed no improved effects on constipation than sham treatment.

There are a number of possible interpretations for these findings. First, moxibustion treatment may truly have had no effect on the treatment of constipation. Second, the small sample size may have contributed to the lack of an observed effect even with a large SD [27]. Third, the moxibustion treatment design may not have been optimal for the treatment of constipation in terms of acupuncture points, treatment frequency, and the number of treatment sessions. Fourth, the sham treatment itself may have had unexpected effects on the treatment of constipation rather than no effect at all. Though there is a study validating this method of sham treatment [21], one subject in the sham group reported rubefaction as adverse event in our trial.

In practice, the treatment effect of moxibustion consists of many factors, such as heat stimulation, the ingredients of the moxa pillar, moxa smoke and the touch sensation when moxibustion is applied on the acupuncture points. Even though the moxa pillar was blocked in the sham treatment to prevent the delivery of heat and the ingredients of the moxa pillar, the touch sensation on acupoints and the smoke from the pillar could have had some positive or placebo effects.

In Oriental medicine, the diagnosis and treatment of a patient is dependent on the condition and symptoms of the patient. Even if patients have the same disease, they may be treated differently based on their syndrome pattern. This differentiation of syndromes is an important characteristic of Oriental medicine [28].

The subjects of this study with an excess syndrome were diagnosed as having excess-cold syndrome. Usually, a deficiency syndrome accompanies a cold syndrome, and an excess one accompanies a heat syndrome. But some subjects are diagnosed as excess-cold by syndrome pattern diagnosis. Moxibustion treatment might have differing roles according to the syndrome pattern. However, the sample size was too small to evaluate the effect of the syndrome pattern.

In addition, small sample could cause type II error, so it could underestimate the effect of moxibustion. Further studies are warranted with larger sample sizes and more clear criteria for excess and deficiency syndromes. Another shortcoming is we did not check whether blinding was successful or not, though this sham blinding method was well established as blinded to the participants in a previous study [21]. Also, Assessors were not blinded, because CRCs evaluated the change of defecation frequency, BSS, and CAS scores, as assessor.

In our study, it could make it difficult to apply these results to the general population of constipated patients because all participants are female. However, it could also strengthen the validity of the differences between the treatment and control groups due to the lack of a gender bias.

Previously reported adverse events related to moxibustion treatment include burns, an itching sensation, infection, allergy and xerophthalmia [29]. In patients with constipation, a previous RCT reported itching, skin eruption, and stinging eyes from the smoke as adverse events [15]. All adverse events in this study were rubefaction with or without itching and were reported as mild.

This study is the first sham-controlled trial using moxibustion for functional constipation. However, it has several limitations as follows: small sample size, criteria for syndrome differentiation, patient and assessor blinding. Further studies with large sample size and blinding are needed. Also, more rigorous and validated sham moxibustion should be developed.

## Conclusion

In conclusion, moxibustion treatment did not present statistically significant effects when compared to sham treatment in terms of the defecation frequency, the stool form and subjective constipation symptoms. More rigorous studies with a larger sample size are needed to verify if there is an effect of moxibustion on constipation.

## Competing interests

The authors declare that they have no competing interests.

## Authors' contributions

JEP designed the study protocol, drafted the manuscript and participated in the study as a coordinator. JUS participated in the study design and conducted the moxibustion treatment. KYK conducted statistical analysis. BCS, KEH and SMC participated in the study design, drafted and reviewed the manuscript. All authors read and approved the final manuscript.

## Pre-publication history

The pre-publication history for this paper can be accessed here:

http://www.biomedcentral.com/1472-6882/11/124/prepub
